# Rationale and design of the RESOLVE trial: lanreotide as a volume reducing treatment for polycystic livers in patients with autosomal dominant polycystic kidney disease

**DOI:** 10.1186/1471-2369-13-17

**Published:** 2012-04-04

**Authors:** Tom JG Gevers, Melissa Chrispijn, Jack FM Wetzels, Joost PH Drenth

**Affiliations:** 1 Department of Gastroenterology and Hepatology, Radboud University Nijmegen Medical Center, Nijmegen, the Netherlands; 2 Department of Nephrology, Radboud University Nijmegen Medical Center, Nijmegen, the Netherlands

**Keywords:** ADPKD, Urinary biomarkers, Polycystic liver disease, Lanreotide

## Abstract

**Background:**

A large proportion of patients with autosomal dominant polycystic kidney disease (ADPKD) suffers from polycystic liver disease. Symptoms arise when liver volume increases. The somatostatin analogue lanreotide has proven to reduce liver volume in patients with polycystic liver disease. However, this study also included patients with isolated polycystic liver disease (PCLD). The RESOLVE trial aims to assess the efficacy of lanreotide treatment in ADPKD patients with symptomatic polycystic livers. In this study we present the design of the RESOLVE trial.

**Methods/design:**

This open-label clinical trial evaluates the effect of 6 months of lanreotide in ADPKD patients with symptomatic polycystic livers. Primary outcome is change in liver volume determined by computerised tomography-volumetry. Secondary outcomes are changes in total kidney volume, kidney intermediate volume and renal function. Furthermore, urinary (NGAL, α1-microglobulin, KIM-1, H-FABP, MCP-1) and serum (fibroblast growth factor 23) biomarkers associated with ADPKD disease severity are assessed to investigate whether these biomarkers predict treatment responses to lanreotide. Moreover, safety and tolerability of the drug in ADPKD patients will be assessed.

**Discussion:**

We anticipate that lanreotide is an effective therapeutic option for ADPKD patients with symptomatic polycystic livers and that this trial aids in the identification of patient related factors that predict treatment response.

**Trial registration number:**

Clinical trials.gov NCT01354405

## Background

Autosomal dominant polycystic kidney disease (ADPKD) most often presents a kidney phenotype with hypertension and renal failure due to continuous growth of renal cysts. It affects all ethnic groups and has an incidence of 1:500 to 1:1000 [1]. ADPKD is inherited in an autosomal dominant fashion, and so far two genes, *PKD1 *and *PKD2*, have been implicated to cause the disease [2].

A large proportion of ADPKD patients suffers from polycystic liver disease, often while renal function capacity is preserved [3] The natural course of polycystic liver disease dictates a continuous progression of size and number of hepatic cysts [4,5]. The rate of progression is still unknown at his time, but recent trials showed that liver volume increases with 0.9-1.6% within one year [6-8].

Most ADPKD patients with polycystic liver disease are asymptomatic until significant hepatomegaly develops. Subsequently mechanical complaints such as abdominal distension, pain, and early satiety arise [9,10]. Other complications include intracystic hemorrhage or rupture of cysts causing acute abdominal pain. Currently available treatment options aim at reduction of liver volume and are mainly surgical [11]. However, drawbacks of surgical therapy are the partial effectiveness, their inherent morbidity and mortality and their inability to change the natural course of the disease.

This has led to the introduction of somatostatin analogues as a medical treatment option for polycystic liver disease. Somatostatin analogues, such as lanreotide and octreotide, are thought to decrease polycystic liver volume through their virtue of cAMP repression [12,13]. We recently performed a trial with the somatostatin analogue lanreotide in polycystic liver patients with autosomal dominant polycystic liver disease (PCLD; isolated polycystic liver disease) or ADPKD [8]. In this trial, 54 patients were randomly assigned to lanreotide or placebo and treated for 6 months. Lanreotide decreased liver volume with 2.9%, while it increased by 1.6% with placebo. Moreover, there was a trend of delayed growth of polycystic kidney volume in the 32 ADPKD patients that participated. Others trials showed similar effects in reducing polycystic liver volume with octreotide [6,7,14]. These observations clearly support the thesis that polycystic liver volume can be reduced by somatostatin analogues.

However, the majority of these trials included a mixture of ADPKD and PCLD patients. It is still unknown if these patient groups have divergent responses to treatment with somatostatin analogues. To eliminate this possible confounding factor, we have designed and initiated a clinical trial (RESOLVE trial) to examine the effectiveness (change in total liver volume) of lanreotide in ADPKD patients with polycystic livers. In addition, we will determine the effect of lanreotide on change in total kidney and kidney intermediate volume. Intermediate volume is tightly correlated with glomerular filtration rate (GFR) and its long-term decline, and may represent a marker for ADPKD progression [15]. Finally, multiple sets of urinary (NGAL, α1-microglobulin, KIM-1, H-FABP, MCP-1) and plasma (fibroblast growth factor 23 (FGF23)) biomarkers have been discovered that are correlated to parameters of ADPKD disease severity, and may be associated with treatment response to lanreotide [16,17]. In conclusion, using the dataset that is generated by the RESOLVE trial, we want to assess (1) whether lanreotide has a beneficial effect on growth of polycystic liver volume, (2) on growth of total kidney and intermediate volume, (3) on renal function, and (4) whether the suggested biomarkers predict treatment responses to lanreotide.

## Methods/design

### Study aim

**Figure 1:**
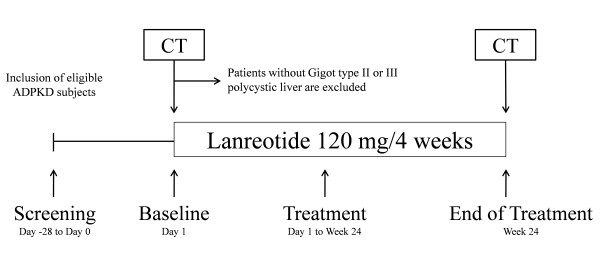
**RESOLVE trial profile**. ADPDK subjects are screened for eligibility, and 43 ADPKD patients with symptomatic polycystic liver disease and eGFR > 30 ml/min (MDRD formula) will be included. At day 1, all patients undergo CT volumetry of liver and kidneys. Patients without polycystic type II or III livers, as determined with CT volumetry, will be excluded from the study. Interval visits are scheduled 4 and 12 weeks after start of treatment. After 24 weeks of treatment with lanreotide (6 injections), another CT will be performed to evaluate the change in liver and kidney volume.

The primary objective of the RESOLVE trial is to determine the effectiveness of lanreotide to attenuate growth of liver volume in ADPKD patients with symptomatic polycystic livers. ADPKD patients with polycystic livers will receive lanreotide 120 mg every 4 weeks for a total of 24 weeks (Figure 1). Secondary objectives are to assess the effect of lanreotide treatment on total kidney and kidney intermediate volume, to follow renal function and to identify biomarkers that predict treatment response. Finally, safety and tolerability of lanreotide treatment in ADPKD patients will also be assessed.

### Study population

ADPKD patients with symptomatic polycystic liver disease Gigot type II (diffuse medium-size cysts; hepatic parenchyma preserved) or Gigot type III (massive, diffuse small- and medium-size cysts; little hepatic parenchyma preserved) between 18 and 70 years are eligible for participation of the study [18]. Symptomatic patients are defined as having at least three of the following symptoms:

• Abdominal pain

• Abdominal distension

• Abdominal fullness

• Dyspnoea

• Early Satiety

• Back pain

• Nausea or vomiting

• Anorexia

• Weight loss

• Jaundice

The diagnosis of ADPKD is based upon the Ravine criteria [19]. Furthermore, patients must have an estimated glomerular filtration rate (eGFR) by the Modification of Diet in Renal Disease (MDRD) above 30 ml/min/1.73 m^2 ^[20]. The specific study inclusion and exclusion criteria are listed below.

#### Inclusion criteria

• Age 18 to 70 years

• Adult subjects with a diagnosis of ADPKD and at least a symptomatic polycystic liver Gigot type II[18]

• eGFR > 30 ml/min/1.73 m^2 ^(MDRD formula) at screening

• Subjects are willing and able to comply with the study drug regimen and all other study requirements

• Signed informed consent

#### Exclusion criteria

• History of renal transplantation

• Use of oral contraceptives or estrogen suppletion

• Pregnancy or breastfeeding

• History of cardiac or pulmonary disease, symptomatic gallstones, pancreatitis or diabetes mellitus

• Intervention (aspiration or surgical) targeted at hepatic or renal cysts within three months of baseline

• Treatment with somatostatin analogues within 3 months of baseline

• Mental illness that interferes with the patient ability to comply with the protocol

• Drug or alcohol abuse within one year of baseline

• Increased liver enzymes (2-fold above normal values); exception is an isolated elevated gamma-glutamyltransferase or alkaline phosphatase, which occurs frequently in polycystic liver disease

• Co-medication with known interaction with lanreotide, like cyclosporine

#### Exclusion criteria for the use of iodine based radiocontrast

• Use of nephrotoxic agents like NSAIDs or diuretics within 24 hours before receiving a contrast-enhanced CT-scan

• History of moderate or severe reaction to contrast injection

• History of contrast induced nephropathy

• Treatment with I^131 ^during the course of the trial

• Diagnosis of Morbus Kahler or Morbus Waldenström

*eGFR < 60 ml/min/1.73 m2 (MDRD formula)

### Study design and setting

The RESOLVE trial is a single centre open label study in subjects with ADPKD. The trial aims to enroll 43 ADPKD patients. The trial design is schematically represented in Figure 1. All eligible patients receive lanreotide 120 mg for a total of 24 weeks. At start and end of treatment, total liver and kidney volumes are measured by computer-tomography (CT) with contrast media if eGFR (MDRD) is > 60 ml/min/1.73 m^2^, or without contrast media if eGFR (MDRD) is 30-60 ml/min/1.73 m^2^. In addition, all patients are evaluated at 4 and 12 weeks after start of treatment. Recruitment has started in July 2011, will last until July 2012 and will be completed by July 2013. This study is submitted to clinicaltrials.gov (NCT01354405).

### Trial treatments

All patients will receive long-acting 120 mg Somatuline^® ^(Lanreotide) administered deeply subcutaneously every 4 weeks (28 days for a total duration of 24 weeks). Another trial documented the efficacy and safety of administration of 120 mg lanreotide in patients with polycystic liver disease and demonstrated that this dosage is well tolerated in patients and there were no notable drop-outs [8]. The most common adverse effect are loose, pale and fatty stools which typically start 24 hours after the first injection of lanreotide and lasts for 1-4 days. Pancreas enzyme replacement is prescribed to ameliorate these symptoms if they persist. In case of lanreotide-associated toxicity, the dose will be reduced with 30 mg until symptoms disappear. Lanreotide will be administered at the patient's home by a dedicated nursing team.

### Primary outcome

The primary outcome of the RESOLVE trial is to assess the effect of lanreotide treatment on total liver volume in ADPKD patients with symptomatic polycystic liver disease. Primary efficacy endpoint is absolute change in liver volume after 24 weeks of treatment with lanreotide compared to baseline liver volume. We compare our findings with the natural course of polycystic liver growth observed in the trials that evaluated somatostatin analogue treatment in PLD patients [6-8].

### Secondary outcomes

The proportional change in liver volume (normalized as percentage) from inclusion to week 24 will be assessed as a secondary outcome. Furthermore, other secondary outcomes are the change in total kidney volume and that of 3 kidney tissue classes [15]. We will distinguish 3 kidney tissue classes on basis of CT images: cysts, parenchyma, and intermediate volume. Intermediate volume represents regions with contrast enhancement markedly lower than that of vascularized parenchyma tissue but higher than that of cysts, and is correlated to GFR decline [15,21]. In addition, changes in quality of life (assessment with EuroQoL questionnaire) and gastro-intestinal symptoms (using a gastrointestinal symptoms questionnaire) will be measured at baseline and at end of treatment [22,23]. Renal function will be assessed by using estimation equations (MDRD and cystatin C) as well as measured creatinine clearance determined by 24-hour urine collection. The frequency and severity of all reported adverse events will be recorded at every visit to evaluate the safety and tolerability of treatment with lanreotide. Finally, urinary biomarkers (NGAL, α1-microglobulin, KIM-1, H-FABP, MCP-1) and serum FGF23 will be assessed before and after treatment with lanreotide [16,17]. These biomarkers are correlated to parameters of ADPKD disease severity and may predict treatment response to lanreotide. As concentration of FGF23 in serum is dependent of vitamin D, parathyroid hormone (PTH), calcium and phosphate serum levels, these parameters will also be measured at start and end of treatment.

### Data collection

Data will be collected into a case record form designed to capture all visit information including medical history, results from laboratory analysis and adverse events. Study duration for all patients is 25-28 weeks in total, divided in a 1-4 weeks screening phase and a 24 weeks follow up phase after start of lanreotide (Figure 1). At screening, a pregnancy test is performed (for women with childbearing potential). Within the follow up phase of the trial, patients are seen at week 0, 4, 12 and 24. During each visit, medical history, adverse events, tolerability and drug accountability are assessed. In addition, vital signs and weight are measured, blood samples are drawn and the eGFR (MDRD) is estimated. Two main study visits including CT volumetry (procedure described below), questionnaires, 24 hour and spot urine collection will take place at start (week 0) and end of treatment (week 24). Furthermore, blood and urine samples will be taken for assessment of serum FGF23, PTH, Vitamin D, Cystatin C and the urinary biomarkers. The requested parameters at the different visits are listed below.

#### Screening

• Written informed consent

• Eligibility criteria check

• Estimated GFR (MDRD)

• General characteristics

• Concomitant therapy and medical history

• Physical examination and vital signs

• Laboratory tests: haematology, biochemistry and lipid profile

• Pregnancy test in females between 18-50 years

#### Baseline (week 0) and end-of-treatment (week 24)

• CT liver and kidney volumetry

• Estimated GFR (MDRD and Cystatin C clearance)

• Creatinine (24-hour urine)

• Urinary biomarkers: NGAL, α1-microglobulin, KIM-1, H-FABP, MCP-1 and creatinine (spot urine)

• FGF23 (serum and spot urine)

• Vitamin D, PTH, calcium and phosphate (serum)

• GI symptom questionnaire

• EuroQol questionnaire

#### Every visit

• Adverse events and concomitant therapy

• Drug accountability

• Physical examination and vital signs

• Weight

• Estimated GFR (MDRD)

• Laboratory tests: hematology, biochemistry and lipid profile

### CT Scanning and 3-Dimensional Volumetry

CT scans at baseline and week 24 will be performed on a multidetector CT scanner (Somatom Sensation 16 or 64; Siemens Medical Solution AG, Erlangen, Germany). All CT scans are blinded to patient identity and date of birth as well as date of scan. The effect of lanreotide will be evaluated by 3D total liver and kidney volume measurement of CT scan slices using Pinnacle^3® ^version 8.0 g (Philips, Eindhoven, The Netherlands). Imaging protocol includes that CT scans have a slice thickness of 3 mm, and liver and separate kidneys will be outlined manually every 9 mm. The software interpolates the intermediate slices and calculates the areas within the indicated circumference, and finally, total liver or kidney volume. The vessels and the ureter in the area of the renal hilum are excluded from manual volumetric marking. Unblinding of CT scans will be performed after all liver and kidney volumes are measured.

### Intermediate volume identification on CT images

Intermediate volume will be assessed as described earlier [21]. Briefly, the kidneys will be outlined manually on all acquired digital images using interactive image editing software (GIMP; GNU Image Manipulation Software, http://www.gimp.org). Subsequently, as an image enhancement step, anisotropic diffusion filtering will be used to smooth high-frequency noise [21]. Binary masks generated from the image outlines will be applied to the enhanced images, and image segmentation will be applied to the resulting kidney regions using a statistical approach known as Otsu's thresholding [24]. After the application of Otsu's method with a number of classes equal to 4, each voxel in the volume will classified as fat, cyst, intermediate, or parenchyma. From the segmented images, cyst, intermediate, and parenchymal volumes will be computed by multiplying the voxel count of each class by voxel volume, as determined by the acquisition protocol [21]. Validation of the segmentation procedure is described previously [21].

### Study withdrawal

Patients will be withdrawn from the study for any of the following reasons: withdrawal of informed consent, pregnancy, failure to adherence to protocol requirements, unacceptable toxicity, surgical intervention during the trial and if the investigators conclude that it is in the patient's best interest for any reason. There will be no option of replacement into the study after withdrawal.

### Sample size considerations

A previous trial suggested that 6-month treatment with lanreotide induced a 134 ml decrease in total liver volume in polycystic liver patients [8]. For the purpose of this study we assumed that lanreotide is able to resort in a similar effect in patients with exclusively ADPKD. A sample size of 39 will achieve 80% power to detect a difference of 150.0 mL (SD 325.0 ml) in liver volume (pre versus after treatment) using a two-sided α-level of 0.05. Taken into account a dropout rate of 10%, the sample size has to be 43 for the complete cohort.

### Statistical analysis

All outcomes will be analyzed on an intention-to-treat basis. Parallel analyses conducted on per-protocol population will be performed. The volume of the liver will be determined as mentioned before. For primary and secondary endpoints, absolute and relative differences between baseline and end of treatment will be analyzed using a paired two-sided t-test, or Wilcoxon ranked sum test where appropriate. All statistical analyses will be two-sided with a critical significance level of 5%.

To evaluate which biomarkers predict treatment response to lanreotide, the association between each biomarker (NGAL, α1-microglobulin, KIM-1, H-FABP, MCP-1 and FGF23) and each of the primary and secondary outcomes will be examined by univariate linear regression analyses. Predictors that are univariately associated with the outcome (*p*-value < 0.10) will be included in multivariate linear regression analyses. The model will be reduced by excluding predictors from the model with a *p*-value of > 0.05. In addition, the following variables will also be included in univariate models as predictors of favorable outcome as a secondary analysis: age, baseline liver volume, baseline kidney volume, kidney intermediate volume and estimated renal function (MDRD and cystatin C). All abnormal laboratory results will be listed and frequency tables will be compiled for Adverse Events classified according to the standard WHO-ART Body System Dictionary and preferred terms.

### Ethical considerations

Ethical approval has been obtained from the local ethics committee of the Radboud University Nijmegen Medical Center. This study will be performed in accordance with the protocol, the guidelines of Good Clinical Practice/ICH, the principles of the Declaration of Helsinki 1964 as modified by the 52^nd ^WMA General Assembly, Edinburgh, Scotland, October 2000 including two notes of clarification paragraph 29 and 30, and the local national laws governing the conduct of clinical research studies. All subjects have the right to withdraw from the study at any time during the trial. Safety of trial subjects is monitored by an independent data safety monitoring board (DMSB).

## Discussion

Treatment with lanreotide may results in several identifiable benefits for ADPKD patients with polycystic liver disease. It may map the road to a causal therapy for patients with polycystic liver disease. Current surgical procedures carry the risk of considerable morbidity and not all patients are qualified for this approach. Alternative options are needed and our trial will establish whether and to which extent and in whom lanreotide treatment can reduce liver volume growth in ADPKD.

The main strength of the RESOLVE trial is to determine whether lanreotide decreases liver volume in patients with ADPKD. Another group performed a post-hoc analysis of data from a randomized, cross-over study in 12 patients with ADPKD [25]. In this analysis, they evaluated the effect of octreotide in 12 ADPKD patients with polycystic livers and found a beneficial effect on liver volume [6]. However, these findings must be taken with caution due to the small sample size and because carry over effects cannot be excluded given the cross-over design. This will be the first trial that is powered to detect a small but significant change in polycystic liver volume in ADPKD patients treated with lanreotide. Furthermore, although we predetermined renal volume and renal function as a secondary outcome, the inclusion of exclusively ADPKD patients allows us to evaluate an effect of lanreotide on kidney volume and function. Third, intermediate volume is tightly connected to the decline in GFR and might be useful as a marker for ADPKD progression [15]. In this trial, we will investigate whether lanreotide has a beneficial effect on intermediate volume. Fourth, it is still unknown which patient factors predict treatment response to somatostatin analogues. Urinary and serum biomarkers associated with ADPKD disease severity are assessed in the RESOLVE trial at baseline and at end of treatment [16,17]. We hope to correlate these markers to treatment response as this will allow us to give evidence based recommendations which ADPKD patients will benefit specifically from treatment with lanreotide. Finally, this design provides an opportunity to study the safety of lanreotide in ADPKD patients with symptomatic polycystic liver disease.

There are limitations that come with our study worth addressing. First, as we seek polycystic liver targeting, ADPKD patients with symptomatic polycystic livers and only mildly enlarged polycystic kidneys are not excluded from the RESOLVE trial. To properly evaluate the effect of lanreotide on polycystic kidney volume, we should have introduced a minimal threshold of total kidney volume. Second, we do not include a control arm in our trial, but rather sought comparison with values at baseline. This prevents us from direct comparison with untreated ADPKD patients. However, based on our observations and the data from placebo arms of other randomized clinical trials, it is possible to establish the natural course of symptomatic polycystic liver disease. In our original trial, an increase of 1.6% in liver volume was observed after 6 months, while in another trial liver volume increased with 0.9% after 12 months [7,8]. Both these trials included a mixture of APDKD and PCLD patients. Liver volume increased with 1.2% during 6 months in a third trial that included exclusively ADPKD patients [6]. As the natural growth pattern of polycystic liver disease does not seem to differ between ADPKD and PCLD, we may use the data from these placebo arms to evaluate if lanreotide affects the growth of polycystic liver volume. In addition, the lack of a control group guarantees that a possible effective therapy is not withheld from symptomatic PLD patients included in the trial, and that the results will be directly applicable to ADPKD patients in the daily practice.

Despite these limitations, our study will add valuable information to the literature of medical treatment of polycystic liver disease.

In conclusion, by designing the RESOLVE trial, we anticipate that lanreotide is an effective therapeutic option for ADPKD patients with symptomatic polycystic livers, and we hope to identify patient related factors that predict treatment response.

## Abbreviations

ADPKD: Autosomal dominant polycystic kidney disease; PCLD: autosomal dominant polycystic liver disease; GFR: glomerular filtration rate; FGF23: fibroblast growth factor 23; eGFR: estimated glomerular filtration rate (eGFR); MDRD: Modification of Diet in Renal Disease; PTH: parathyroid hormone; CT: computer-tomography; DSMB: data safety monitoring board

## Conflict of interests

The authors declare that they have no competing interests.

## Authors' contributions

TG, carries out the trial, performs biomarker analyses and drafted the manuscript. MC, participated in the design and coordination of the study, and performed statistical analysis. JW, participated in the design of the study and revised the manuscript critically for important intellectual input. JD, conceived of the study, and participated in its design and coordination. JD helped to draft the manuscript and gave final approval of the version to be published. All authors read and approved the final manuscript.

## Pre-publication history

The pre-publication history for this paper can be accessed here:

http://www.biomedcentral.com/1471-2369/13/17/prepub
